# Promoting planetary health and well-being for all: harnessing indigenous knowledges for health with traditional, complementary and integrative health systems

**DOI:** 10.3389/fmed.2025.1543687

**Published:** 2025-04-08

**Authors:** Georg Seifert, Hiba Boujnah, Roshanak Ghods, Susan Wieland, Obijiofor Aginam, Anchalee Chuthaputti, Ricardo Ghelman, Cheng Soon Goh, Motlalepula Gilbert Matsabisa, Sione Tu'itahi, Sungchol Kim, Shyama Kuruvilla, Bhushan Patwardhan

**Affiliations:** ^1^Charité Center for Traditional and Integrative Medicine (CCCTIM), Berlin, Germany; ^2^Charité University Medicine Berlin, Berlin, Germany; ^3^Department of Traditional Medicine, Institute for Studies in Medical History Persian and Complementary Medicine, Tehran, Iran; ^4^School of Medicine, University of Maryland, Baltimore, MD, United States; ^5^United Nations University-International Institute for Global Health, Kuala Lumpur, Malaysia; ^6^Department of Thai Traditional and Alternaive Medicine, Ministry of Public Health Thailand, Mueang Nonthaburi, Nonthaburi, Thailand; ^7^Department of Medicine on Primary Healthcare of Federal University of Rio de Janeiro Medical School, Rio de Janiero, Brazil; ^8^Traditional and Complementary Medicine Division, Ministry of Health Malaysia, Kuala Lumpur, Malaysia; ^9^University of the Free State, Bloemfontein, South Africa; ^10^Health Promotion Forum of New Zealand, Wellington, New Zealand; ^11^World Health Organization, Geneva, Switzerland; ^12^Savitribai Phule Pune University, Pune, India

**Keywords:** health, Indigenous Knowledges, planetary health, Traditional, Complementary, and Integrative Health, well-being

## Abstract

The concept of well-being extends beyond individual health practices to encompass a burgeoning industry focusing on healthy lifestyles and products. This trend reflects a global paradigm shift toward prioritizing the holistic well-being of individuals and the planet within socioeconomic policies. This shift underscores the integration of social, economic, and environmental considerations into policy frameworks, signaling a concerted effort toward a more sustainable and health-conscious future. The article highlights the crucial role of Indigenous Knowledges for Health (IKH) and Traditional, Complementary and Integrative Health/Medicine (TCIH) systems in contributing to the interconnectedness between human well-being and the health of our planet through innovation, health promotion, and fair, equitable and sustainable benefit sharing. Various contemporary global problems stem from ways of thinking that prioritize the short-term economic interests of individuals or specific groups over the well-being of Peoples and the planet as a whole. In contrast, IKH and TCIH systems often inherently adopt a holistic, sustainable worldview where individual, community, and Planetary Health and well-being are intertwined, providing transformative solutions and models toward integrative health. By incorporating IKH and TCIH systems into contemporary development models, health, and medicine, we can promote health equity, improve well-being, and create a sustainable future for Peoples and the planet.

## Highlights

- There is an emerging consensus to move beyond current conventional health care models and develop a broader understanding of health and well-being to meet Sustainable Development Goals (SDGs).
- Health is essential to overall well-being but it is not the sole determinant of well-being.
- Incorporating measurable benchmarks of health that align with the holistic principles of IKH and TCIH systems can expand the scope of proposed well-being frameworks.
- The concepts of Planetary Health and One Health are not new, as they have been fundamental to ancient cultures, traditions, and Indigenous communities for centuries.
- The philosophies and practices that underpin IKH and TCIH systems can lead to innovative ideas and novel research in the field of health and well-being.
- Personalized medicine and holistic health, drug discovery and pharmacology, autophagy and fasting, chronobiology and lifestyle, psycho-neuro-immunology and mind–body approaches, and Anthropocene and nature-based practices are a few examples of links with IKH and TCIH.
- Ensure protection of Intellectual Property (IP), access to knowledge, benefit sharing, and preservation of genetic resources.
- Integrating conventional health and development systems with IKH and TCIH systems could contribute to achieving the SDGs and ultimately to ensuring the health and well-being of Peoples and the planet.

## Introduction

Some of the major human-induced global challenges presently converging and confronting humanity include geo-political conflicts, environmental catastrophes, notably climate change, economic crises, and global health emergencies. Recently, the COVID-19 pandemic and multiple natural disasters linked to climate change have highlighted the interdependence of human health and the health of the planet, exposing the fragility not only of health systems but of entire societies and ecosystems throughout the world. These events have also exacerbated global inequities and further exposed the vulnerability of marginalized groups within society, accentuating the vital importance of social trust and cohesion. The subsequent social, economic, and political disruptions have pushed decision-makers to rethink their present intellectual property and commercialization models built on profit at the expense of Peoples and the planet.

### The current policy landscape of traditional medicine: momentum to be seized

The Helsinki Declaration 2020 recognized a strong link between human and Planetary Health and emphasized the need to prioritize addressing environmental calamities and social justice.

It is also recognized that scientific insights and innovations are more effectively translated into action through a systemic approach, fostering collaboration between scientists, societies, and policymakers (1)

Currently, a broader understanding of health and well-being is essential for developing comprehensive strategies and policies that promote a holistic approach to individual, populations, and Planetary Health. This requires collaboration between individuals, communities, health care systems, policymakers, and other actors to create environments that enable individuals to lead healthier lives on a healthy planet.

In 2023 the 76th World Health Assembly (WHA) passed the decision to develop a new World Health organization (WHO) global strategy for Traditional Medicine. Developing a new WHO strategy provides a new strategic opportunity to harness the potential and contributions of traditional medicine in health promotion, disease prevention and well-being of Peoples, and Planetary Health and well-being. The WHO strategy, currently under development, would also acknowledge the need to reorient health systems on primary health care as a foundation for Universal Health Coverage (UHC), health security, and a global well-being framework focused on Indigenous Peoples’ health. This 3^rd^ global strategy succeeds the two previous strategies endorsed in 2002 and 2014, respectively. These earlier strategies significantly advanced the standardization of terminologies, training methodologies, and practices within diverse traditional medicine systems. Additionally, they played a pivotal role in ensuring the quality and safety of traditional medicine services (2). The latter strategy has further emphasized the principles of the Alma-Ata Declaration 1978 and Astana Declaration 2018. Concurrently, the final report of the WHO Council on the Economics of Health for All recommended the co-creation and reshaping of an economy that delivers on the goals that are critical to human and Planetary Health and well-being. This report discussed the interlinkages between the fields of economy, the human health, and Planetary Health. It further reconsidered the dynamic nature of concepts related to health and well-being and advocated for a critical refocusing on Planetary Health, health promotion, primary health care, integrative medical care, public health, and Planetary Health overall.

Traditional, Complementary, and Integrative Health (TCIH) has emerged as a worldwide phenomenon not only in some developing countries but also in industrialized countries. The demand for alternative approaches to health is increasing as patients strive for greater autonomy in managing their health and well-being, expressing a preference for health care that is both compassionate and customized to their individual needs. In contrast to this trend, for most of the global population, especially those in remote and rural areas, TCIH is the preferred and sometimes only option for maintaining health and well-being. This approach to health care provides culturally acceptable, accessible and affordable, and above all resource-efficient and sustainable care and motivates the WHO to strategically support TCIH worldwide. In August 2023, the WHO and the Ministry of Ayush, Government of India, co-hosted the Traditional Medicine Global Summit in Gandhinagar, aligning with India’s G20 Presidency. Organized alongside the G20 Health Ministerial Meeting, the summit aimed to secure political support and evidence-based initiatives for TCIH. The “Gujarat Declaration,” the outcome document of the summit, reaffirms global commitments to Indigenous Knowledges for Health (IKH), biodiversity, and TCIH (3). Further, the declaration underscores the importance of applying rigorous scientific methods for a more comprehensive, context-specific, and personalized approach to health and well-being worldwide (3).

### Redefining health and well-being in the Anthropocene

We are living in the ‘Anthropocene’ epoch, a term coined to recognize that all human actions continue to profoundly change the Earth system, and the global human-made mass is now reported to exceed all living biomass (4). Planetary transformation, including changes to the atmosphere, climate, ecosystems, and biodiversity, has enormous and deeply disturbing implications for human health especially in low-income settings (5).

Recognizing the inherent interconnectedness of human activities, the state of the planet, and the well-being of both humans and the environment, the United Nations Development Programme (UNDP) has proposed an experimental index that adjusts the Human Development Index (HDI) to account for Planetary Pressures in the Anthropocene, known as the Planetary Human Development Index (PHDI) (6). Concurrently, the WHO Council on the Economics of Health for All report has recommended the co-creation and reshaping of economies in a manner that considers both human and planetary well-being with Health for All as the primary goal (7). Moreover, the 2030 Agenda and SDGs explicitly articulate a transformative blueprint for action encompassing the spheres of Peoples, the planet, and prosperity.

Against this background, it is necessary to move beyond conventional economic measures like Gross Domestic Product (GDP) to consider measures such as the green GDP, which subtracts environmental and social costs from the conventional GDP and embrace measures like the Gross Ecosystem Product (GEP) (8). GEP considers the value of nature’s contributions to economic activity and measures how much ecosystems contribute to human well-being (9, 10). The GEP considers not just goods and services but also the value of nature, citizenry, equal opportunity, safety, peace, and well-being.

The conventional health care system often focuses on treating specific symptoms or diseases rather than the whole person. In contrast, IKH and TCIH systems are rooted in diverse cultural practices that address the whole person within a social, natural, and spiritual context, and can offer insights and solutions for more inclusive and better health care to serve diverse communities. It is important to establish mechanisms for collaboration and integration between TM and conventional health care systems leveraging the potential of IKH toward TCIH. This should involve research, education, policy development, and regulation to ensure safety, efficacy, and quality standards. This can enhance the overall effectiveness, cultural relevance, and accessibility of health care services, leading to improved health outcomes for individuals, communities, and societies, while supporting the planet’s health.

TCIH includes various healing systems, healthy lifestyles, mind–body medicine, herbal medicines, and other health-promotive practices that are also rooted in Indigenous Knowledges for Health (hereinafter referred to as IKH and TCIH) systems (11). While we use the terms TCIH and IKH in tandem for the purpose of this discussion, it is imperative to recognize that this amalgamation does not seek to oversimplify the nuanced differences inherent in these concepts. Rather, it serves to shed light on the commonalities that exist in their holistic approaches to health, acknowledging the shared emphasis on interconnectedness, cultural relevance, and a profound understanding of the reciprocal relationship between human well-being and the environment.

The IKH and TCIH approaches need to be transformed through scientific strategies and transdisciplinary collaboration with the precision of frontiers of science such as molecular biology and epidemiology, and incorporating perspectives from physics and medicine to art, to emerge in the future as One Medicine.

As an example of that, studies in Brazil looked at the role of herbal medicine promotion in bioeconomic growth and improved healthcare access and have found that while herbal medicine offers potential for enhancing healthcare accessibility and sustainable livelihoods in tropical forests, this potential remains contingent upon overcoming regulatory, institutional, and socioeconomic barriers in Brazil (12). This underscores the importance of holistic strategies that harness IKH and TCIH while addressing complex socio-economic and environmental challenges, advancing human and planetary health.

Therefore, establishing a culture of transformation begins with Integrative Development Goals (IDGs), which start at the individual level but can be applied in perspective at four levels: the individual, the community, society, and the planet (13). Achieving this goal requires a full understanding of the various dimensions of the human being, including body, soul, and spirit, and their needs. This knowledge is contained in many IKH and TCIH systems, such as ethno-religious traditions and recommendations for health and longevity, which are designed to simultaneously promote the health of the individual, the community, and the planet. Strengthening individual health and radiance thus contributes significantly to the achievement of the SDGs.

#### Evolving concepts of health and well-being

The etymological meaning of health is related to a complete thing. Health encompasses various aspects such as prosperity, happiness, resilience, welfare, preservation, safety, and salutation (14). The WHO defines health as a state of complete physical, mental, and social well-being and not merely the absence of disease or infirmity (80). Health is also considered the ability to adapt to the environment empowering individuals to be agile for well-being and a more compassionate, comforting, and creative approach toward personalized medicine Editorial (15). The salutogenic concept of health emphasizes global well-being concerning inherent healing, stress-managing capacity, coping, and a sense of coherence (16).

Health is an essential component of overall well-being, but it is not the only determinant. SDG 3 aims to ensure good health and well-being for all at all ages. This is also linked to the co-benefits for addressing other SDGs, including poverty reduction, ending hunger, education, social protection, economic development, urban development, etc. The idea of well-being goes beyond the mere absence of illness and includes the promotion of positive health outcomes and quality of life. Several frameworks and measurements for well-being were developed to capture its multidimensional nature. A meta-analysis of longitudinal studies in the general population has suggested that subjective well-being is associated with a decreased risk of mortality (17). Children, as a particularly vulnerable group, need to be given special attention worldwide, as promoting well-being at a young age protects against various chronic diseases in adulthood (18). Well-being is measured by assessing people’s perceptions: feeling healthy, satisfied, or content with life, the quality of relationships, positive emotions, resilience, and realization of their potential. The assessment of functional limitations also helps in determining the Health-Related Quality of Life (HR-QoL).

#### From health and health care to planetary health

The terms ‘health’ and ‘medicine’ are used many times interchangeably, as also ‘health care’ and ‘medical care’. Typically, medicine is defined as the science and art dealing with the maintenance of health and the prevention, alleviation, or cure of disease (Dictionary, n.d.). Based on the current understanding of health, medicine should address well-being in addition to disease or infirmity. However, the current notion of health care centers predominantly around medical care, primarily dealing with symptoms, disease, diagnosis, and treatments. Health care has less focus on prevention and health promotion, and even less engagement in supporting and optimizing well-being within a social and environmental context. Current trends have shown that people are looking for pluralistic health models to promote health beyond conventional medical management (19).

Health results throughout the course of life when individuals’ resources – and social and environmental determinants – suffice to respond satisfactorily to the demands of life (20). A life-course approach to health and well-being encompasses strategies across individuals’ lives that optimize their functional ability, considering the interdependence of individual, social, environmental, temporal, and intergenerational factors, thereby enabling well-being and the realization of rights (21). Health in All Policies has been evolving for about 20 years and recognizes that improving health outcomes can result in tangible co-benefits for other sectors (22).

In recent years, it has become increasingly evident how interconnected Planetary Health, public health and individual health are. Individual health and healthy social conditions are a prerequisite for Planetary Health and vice versa (23).

The physical, mental, and social functioning of people are not the only factors determining human health and well-being. The emerging field of Planetary Health aims to understand how environmental changes in our ecological life support systems threaten our health and why protecting nature is the only way to protect ourselves (24). The Rockefeller Foundation–Lancet Commission recognized that health and civilization depend on the wise use of sustainable and balanced natural systems. This gradually evolved into a concept of Planetary Health that goes beyond human health. Planetary Health considers communities, animals, plants, and various ecosystems and highlights the importance of mutual existence for this and future generations (23).

WHO, United Nations Environment Program (UNEP), Food and Agriculture Organization (FAO), and the World Organization for Animal Health (WOAH), are working together to mainstream One Health, in order to be better prepared to prevent, predict, detect, and respond to global health threats and promote sustainable development. One Health is an integrated, unifying approach that aims to sustainably balance and optimize the health of people, animals, and ecosystems. It recognizes the health of humans, domestic and wild animals, plants, and the wider environment are closely linked and interdependent (25). The Planetary Health and One Health strategies endorse the spirit of the WHO Global Strategy on Health, Environment, and Climate Change that should be included in all policies (26).

### IKH and TCIH: relevance and opportunities

IKH and TCIH systems’ philosophies, principles, products, and practices can be sources of innovation and research to catalyze ancient wisdom and modern science to improve the health and well-being of Peoples and the planet. Some of the key features, principles, and practices from IKH and TCIH may provide newer directions and innovative approaches to further SDGs in the right spirit. The next section will highlight how IKH and TCIH systems offer opportunities in policy development (WHO Wellbing Framework) and in linking to concepts like One Health and Planetary Health. We’ll also explore how these practices can positively impact medical research and treatment, providing valuable insights for enhancing modern health care approaches.

#### Well-being frameworks: leveraging IKH and TCIH

The Geneva Charter for Well-being developed by the WHO sets the foundations for a political reorientation and recommends creating sustainable “well-being societies” to achieve equitable health without breaching ecological limits (27). It recommends creating social structures to support people to take control of their lives and health and advocates investments that integrate planetary, societal, community, and individual health and well-being. It also advocates fundamental redirection of societal values and action consistent with the 2030 SDGs. In addition, the World Health Assembly (WHA) considered a global framework with an implementation, and monitoring plan for integrating well-being into public health using the health promotion approach (28). While the Geneva Charter for Well-being calls for immediate political actions to align with key elements of the SDGs and to promote societal well-being (29), the WHO draft Well-being Framework presents strategic directions to promote societal well-being and ties these together with suggested effective policy orientations drawn from the global health community and country-level experience (30).

The WHO well-being frameworks share common principles with Planetary Health, such as universal human rights, social and environmental justice, sustainable development, solidarity, equity, bioethics, gender, inter-generational parity, interculturality, and peace. As a policy concept, well-being centers on the HR-QoL of peoples and communities (including access to education and employment, participation in society, and other relevant indicators), equity, and planetary sustainability. The different patterns of well-being observed across countries may be related to differences in the countries’ GDP, social protection system, economic situation, health care provision, lifestyle behaviors, or living conditions (31).

The strategic directions for well-being proposed in the framework also resonates with Indigenous Knowledges and health-related cultural practices across communities. The well-being framework may be optimized by considering the key hallmarks of health proposed for designing interventions on human health (32). In a recent attempt, a framework based on 50 measurable biomarkers is proposed to objectively assess positive health (33). A robust model based on measurable hallmarks of health linked to the holistic principles of IKH and TCIH systems may expand the horizon and broaden the scope of the proposed framework.

Appropriate use of the available scientific evidence to reframe well-being and mental well-being within a global public health framework alongside Western biomedical practice remains challenging (34). The Global Conference on Primary Health Care (PHC) adopted a broader vision in the Declaration of Astana 2018 focusing on sustainable primary health care and the need to empower individuals and communities (35). IKH and TCIH can play a significant role in achieving the health-related SDGs (36).

#### IKH and TCIH principles for planetary health and one health

The principles behind Planetary Health and One Health are not new, and the Geneva Charter for Well-being underscore the prioritization of Indigenous Knowledges and leadership (27). IKH and TCIH, through ancient cultures, traditions, and Indigenous communities, place a strong emphasis on promoting healthy lifestyles in harmony with societies and natural environments, and recognizing the importance of balance, nutrition, exercise, self-care and spirituality or purpose in maintaining well-being. IKH and TCIH are often based on locally available knowledge, resources and practices, which when reinforced by science can provide accessible, cost-effective, equitable health care options with improved access to marginalized communities. The association between the health of Peoples and natural systems is central to Indigenous cultural practices and spiritual traditions. The importance of living in harmony with nature and promoting an idea of planetary consciousness can provide deep insights to improve health and well-being. IKH and TCIH systems have become more relevant and can offer potential solutions as we progress toward attaining SDGs (37).

Health and well-being according to IKH and TCIH principles are based on the harmony in the interrelationship between the human and the planet ecosystem. Many traditional branches of TCIH offer fascinating definitions of health that take a preventative and sustainable approach with clear future-oriented goals. These systems emphasize the importance of prevention through a balanced lifestyle and a mindful approach to energetic forces for overall well-being. Even if the underlying concepts are not easily explained by scientific models, relevant implications for preventive approaches and health promotion arise from the enormous wealth of traditional empirical knowledge. Ancient Greek philosophy refers to the structural similarity between the human being (the microcosm) and the cosmos as a whole (the macrocosm) (38). The Greek word *eudemonia* means the state or condition of ‘good spirit,’ which can also be translated as well-being. Similar concepts exist in many other philosophical systems, worldwide, including ancient Mesopotamia, Chinese, African, Persian, Indian, and other Asian traditions. An Indigenous concept Waiora from Aotearoa, New Zealand, reflects the wisdom of Indigenous worldviews showing how people’s health and the natural environment are interconnected (39). In most African societies, people resort to ethnomedicinal therapies for healing. African cultural and philosophical worldviews on health and healing are driven by ethno-medicinal conceptions of health as the outcome of a harmonious combination of physical, non-physical, and social factors (40).

Avicenna in his book *Canon of Medicine* viewed health as a state of balance and harmony within the body, where all the bodily processes are functioning correctly according to the individual’s unique characteristics (Avicenna (41)). Ayurveda considers that all anatomical and physiological functions within the human body reflect the activities in the universe. The concepts of positive health, holistic health, good health, and well-being can be best captured in the Ayurveda concept *of swasthya*, meaning “being contented in one’s natural state of inner harmony” or a state when the body, mind, and spirit are in homeostasis and equilibrium resulting comfort, coping, resilience, happiness, and bliss (42). Similar concepts exist in several other cultures and traditions.

### Integrating traditional wisdom: enhancing modern health care approaches

#### Holistic health and personalized medicine

Integrative medicine is viewed as the beginning of a new age of hope in the post-COVID-19 era. The National Health Services (NHS) in England has initiated a new integrative medicine-based “Personalised Care” movement that recognizes the importance of each individual and their choices, beliefs, culture, and history Michael (43). Several emerging approaches based on the whole person, whole health, and personalized medicine resonate with the holistic principles of IKH and TCIH. The US Veterans Health Administration has adopted a Whole Health approach that promotes personalized and patient-centered care, emphasizes self-care, and incorporates integrative approaches (44). Patient Revolution is another movement that aims to empower individuals to be active participants in their own health care decisions and advocates for a person-centered rather than industrialized approach to health care (45).

IKH and TCIH can provide a unique opportunity to explore a new way of evaluating the holistic principle and whole system research. The individual-centered approaches from IKH and TCIH offer a great potential to complement the genomic route to personalized medicine. Recent studies linking biochemical and molecular correlates of Ayurveda Prakriti types and genomics have provided new insights into mind–body typology, pharmacogenomics, phenotype–genotype relationship, and personalized medicine (46, 47). Similar perspectives regarding mind–body typological systems and personalized medicine are also found in other traditions, including Persian medicine (48), Korean Sasang (49), and TCM (50).

#### Modern pharmaceutical discoveries from herbal medicine

Herbal medicine is a key element across IKH and TCIH practices. The reverse pharmacology approach brings together knowledge and experiences from IKH and TCIH and uses a scientific, experiential methodology to expedite the drug discovery process (51). Over 40% of conventional pharmaceutical formulations and landmark drugs, including Metformin, Taxol, Reserpine, Quinine, and Aspirin, have originated from natural sources (52). The Nobel Prize-winning discovery of Artemisinin from TCM for the treatment of Malaria has inspired scientists to reconsider natural product drug discovery from IKH and TCIH sources (53). Several herbal drugs from IKH and TCIH sources, such as Ginseng, Ashwagandha are known to be adaptogens, anti-stress, and immunomodulators (54). In many countries during the Covid-19 pandemic, traditional medicines with a long history of safe and effective use were repurposed with the help of pre-clinical and clinical studies for possible use in COVID-19 management (55, 56). IKH and TCIH-inspired multi-target synergistic formulation development is considered a future drug discovery strategy (52).

#### Therapeutics based on fasting practices

Fasting has been practiced for religious and cultural reasons, health promotion and therapeutic purposes. Autophagy, a molecular mechanism of longevity may be linked to therapeutic fasting. Caloric restriction and fasting have been reported to produce several physiological benefits such as an increase in insulin sensitivity, and regulation of repair mechanisms (57). Therapeutic fasting is claimed to improve cardiometabolic health, be a valuable support in cancer treatments, stimulate stem cell production, and improve neuronal functioning, well-being, and longevity (58).

The evolving field of circadian medicine is transforming the treatment of significant clinical conditions, such as cardiovascular diseases, cancer, infectious diseases, sleep disorders, and mental health conditions (59)^.^ A daily routine based on the natural biological rhythm includes specific diet and lifestyle activities such as eating, working, and sleeping, divided into six spells throughout the day (42).

#### Nature-based interventions

Historically, IKH and TCIH practices are centered around harmony with nature. Nature-based interventions (NBIs) have been increasingly recognized across disciplines from urban planning to medicine as effective strategies for improving health and well-being. These interventions involve activities in which individuals engage with natural environments, for example, groups walking in nature, bathing in the forest, and community gardens in hospitals or parks in cities (60). Contact with nature is associated with improvements in memory, cognition, and attention, reduction in symptoms of depression and anxiety, lower stress levels, reduced levels of stress hormones, blood pressure, and healthy sleep patterns (61).

A concept of forest therapy known as shinrin-yoku is reported to have beneficial physiological and psychosocial effects useful in preventive medicine and stress management (62)^.^ Systematic reviews have also reported a cost-effective positive impact of ‘forest bathing’ on individual quality of life indicating its possible role in improving public health and well-being promotion (63).

#### Practices based on harmonizing body, mind, and spirit

Various techniques of physical postures, breathing, and meditation based on IKH and TCIH may be effective in improving several physical, mental, cognitive behavior and psychosomatic conditions, including musculoskeletal and neuroendocrine disorders, depression, anxiety, stress, insomnia, addiction, psychosis, pain, hypertension, weight control, and cancer-related symptoms (64). The practice of intense mindfulness meditation is reported to help reduce stress, protect the telomeres from shortening, and produce positive psychological change (65).

Various mind–body medicine interventions may be useful in enhancing cell-mediated and mucosal immunity and reducing inflammatory components (66)^.^ Oxygen sensing and adaptability are known to help in the management of anemia, cancer, and many other diseases (67). Pranayama, a Yoga breathing technique for controlling and regulating the breath, has been reported to result in beneficial changes in oxygen consumption and claimed to enhance Nitric Oxide (68). Several traditional breathing techniques, including Buteyko and Reiki from Japan; Tummo from Tibetan Buddhist, Huna an ancient Hawaiian, Tai chi and Qigong from Chinese, combine specific breathing, physical movements, and meditation techniques claimed to enhance energy flow, improve lung function, relaxation, and consciousness (69).

These examples illustrate only the beginning of how sustainable Planetary Health, affordable and equitable health care, and integrative personalized medicine for the future can and should work together to find synergy with resources from IKH and TCIH systems.

### Challenges to integration

IKH and TCIH approaches to health and medicine are participatory and inclusive in harmony with Mother Nature. Systematic integration of biomedical and IKH approaches into the health promotion program has been reported to be beneficial for mental, physical, spiritual, and family well-being. The Fundamentals of Care framework based on the Māori-centred model considers Indigenous worldviews giving due importance to relationships, family, and spirituality on well-being (70). There is an emerging consensus regarding the need for improving health equity, especially among the colonized Indigenous Peoples globally (71).

The holistic approach of IKH and TCIH needs to coexist with the conventional approach to pathology-based discrete health problems. The integration of these principles within national health systems can ensure the public benefits from medical care and also from a larger perspective on health and well-being within the planetary sustainable development context.

#### Epistemology and evidence

Scientific methodologies stemming from traditional medicine can lead efforts to elucidate the potential benefits and limitations of both conventional approaches and, IKH and TCIH practices. Achieving this requires immersion within the respective cultures, prioritizing a nuanced understanding of stakeholders associated with IKH and TCIH. This necessitates acknowledging their distinctive epistemological perspectives and environmental contexts, fostering collaboration with the communities and regions where these concepts and practices originate (72).

Many IKH and TCIH practices are affordable and often technically simple, but their application to the diagnosis and treatment of the individual can be complex. Many of the current adoptions of IKH and TCIH approaches focus on isolated components which are standardized and then tested using randomized controlled trials. However, the dissolving of IKH and TCIH approaches into multiple detached practices does not represent the ontological and epistemological perspectives of traditional systems and overlooks the potential of their full application. The accurate assessment of the potential benefits or harms of whole systems approaches requires the inclusion of research methods based on these different perspectives (73). The simplified assessment of an individual component from an IK and TCIH practice standpoint will not capture the full impact of the component as integrated into the whole practice and applied to an individual in a non-standardized fashion; it has a limited ability to understand and predict the likely effects of the whole system approaches. While the challenges of complex interventions and personalized treatment also exist within conventional medicine, they are particularly acute in TM due to the foundational philosophy of interconnected treatment modalities supporting individualized, person-centered care.

Experimental methods should not be abandoned, rather there is a need to build bridges and create an environment for mutual learning in which the TM-inspired experiential and conventional experimental epistemologies transform each other for the greater good (74). It is vitally important that there is a dialog between the two health approaches and that they are appropriately integrated for society’s well-being.

#### Equity and sustainability

Sustainable development is defined as a development that meets the needs of the present without compromising the ability of future generations to meet their own needs. This resonates well with the IKH and TCIH ethos, which considers the health of humanity and the natural systems and builds it on the principles of holism and interconnectedness, strengthening public health and health promotion action on ecological and social determinants (75). A need to re-establish a new set of values, a renewed sense of spirituality, reverence for nature, and sustainable development has been advocated for improving Planetary Health (39). A new philosophy involving a pluralistic approach to reimagining the present health system by evidence-based integration of TM is considered as an inclusive approach for achieving the health-related SDGs, especially UHC (76) and health and well-being for all at all ages.

### Equity

Ensuring equitable access to and sharing of benefits of IKH and TCIH must be assured in any integrative efforts, including with respect to Intellectual Property rights and profits, and protecting biological diversity. IKH and TCIH interventions mainly depend upon natural sources. IKH and TCIH, and environmental sciences have helped us to recognize and appreciate the value of the natural environment and its fragility. IKH and TCIH also provide innovative ways of awareness and co-existence with natural ecosystems, which modern science should embrace (77). However, the commercial exploitation of IK and.

TCIH has raised concerns regarding Intellectual Property rights. The issue of biopiracy has sparked international discussions and efforts to address the protection of IKH and TCIH.

The WHO recognizes the value of IKH and TCIH and acknowledges the importance of protecting Intellectual Property rights associated with them. The UN SDGs also emphasize the need for equitable access to, and benefit-sharing of IKH and TCIH. International agreements such as the Convention on Biological Diversity (CBD) and the Nagoya Protocol provide a framework for addressing issues related to Intellectual Property and benefit-sharing concerning traditional knowledge and genetic resources (78).

### Sustainability

Planetary Health, individual health, and the principles of TCIH are intricately linked and offer a comprehensive perspective on the well-being of Peoples and the planet. Sustainable health care requires not only the consideration of individual health aspects but also the integration of ecological and social factors. Studies in Indonesia found that to mitigate climate change impacts on Indonesia’s valuable medicinal resources, there is a need to integrate traditional ecological knowledge with contemporary conservation strategies (79). Preserving healthy nature and biodiversity forms the basis for TCIH, and the methods and principles in turn positively impact nature and biodiversity by promoting sustainable lifestyles and resource use. While a healthy planet contributes significantly to healthy humans, it is important to keep the health of the planet at the center while seeking better human health.

The field of TCIH has witnessed substantial increases in the commercialization of medicinal products, posing risks to vulnerable plant and animal species in certain regions due to overharvesting (12). Studies in Brazil showed that the formalization of herbal medicine has marginalized smallholders, traditional knowledge holders, and Brazil’s native biodiversity, underscoring policy failures in fostering inclusive economic participation (12). The vulnerability of medicinal plants and the risk of their loss is further exacerbated by climate change (79).

This trend raises further concerns about the potential replication of errors seen in the pharmaceutical industry, contributing to larger carbon footprints as the TCIH sector experiences increased industrialization and investment. It is not uncommon that TCIH initiatives predominantly benefited urban centers, failing to address the needs of marginalized regions prone to deforestation such as the Brazilian Amazon, where conflicting economic models highlight tensions between extractive practices and inclusive socio-biodiverse economies (12).

This diverges from Indigenous Knowledges for Health (IKH) applications, where monetary value is often not central to the healing experience. Preserving the environment is essential to prevent the recurrence of past mistakes in the health care domain and to maintain the integrity of TCIH as it evolves.

## Conclusion

IKH and TCIH principles connect us to our roots and remind us to respect the continuity of our shared human endeavor for sustainable living and well-being. IKH and TCIH, with their holistic perspectives, could take the lead in forging the way forward to humans benefiting from and using planetary resources wisely and sustainably. The world is seeking sustainable, nature-friendly, cost-effective, accessible, and innovative ways to achieve health and well-being for all. The relevance of IKH and TCIH in the present situation is of high significance because of its dual benefits to both the humans and the planet through a person-centered, holistic health promotion-based approach that empowers people to take control of their health and well-being while also promoting the well-being of the planet. The integration of IKH and TCIH into health care systems can promote a more holistic and sustainable approach to health and well-being. This requires collaboration between individuals, communities, health care systems, policymakers, and other stakeholders to create environments that support and empower individuals to live healthy lives on a healthy planet as a foundation for UHC, health equity, security, and sustainable development. Therefore, it is crucial that policymakers and health care providers prioritize evidence-based integration of IKH and TCIH, frontiers of science and conventional medicine for disease prevention, health promotion, primary care, and the good health and well-being of all Peoples and the planet Earth.

## References

[1] Halonen JI Erhola M Furman E Haahtela T Jousilahti P Barouki R. The Helsinki declaration 2020: Europe that protects. Lancet Planet Heal. (2020) 4:e503–5. doi: 10.1016/S2542-5196(20)30242-4, PMID: 33159874

[2] WHO (2023). Global strategy on traditional medicine. 76th world heal. Assem. Available online at: https://apps.who.int/gb/ebwha/pdf_files/EB152/B152(18)-en.pdf (Accessed July 29, 2023).

[3] WHO (2023). WHO traditional medicine global summit 2023 meeting report. In Gujarat declaration (Gandhinagar, India: World Health Organization, Geneva), 4. Available online at: https://www.who.int/publications/m/item/who-traditional-medicine-summit-2023-meeting-report--gujarat-declaration

[4] Elhacham E Ben-Uri L Grozovski J Bar-On YM Milo R. Global human-made mass exceeds all living biomass. Nature. (2020) 588:442–4. doi: 10.1038/s41586-020-3010-5, PMID: 33299177

[5] Butler CD. Sounding the alarm: health in the Anthropocene. Int J Environ Res Public Health. (2016) 13:665. doi: 10.3390/ijerph13070665, PMID: 27376314 PMC4962206

[6] Conceição P. Human development report 2020-the next frontier: Human development and the Anthropocene. United Nations Dev. Program. Hum. Dev. Rep. (2020)

[7] Mazzucato M. Health for all: transforming economies to deliver what matters. BMJ. (2023) 381. doi: 10.1136/bmj.p1175 37220921

[8] Rauch JN Chi YF. The plight of green GDP in China. Consilience. (2010):102–16.

[9] Linbo Z Chaozhi H Yang S Yiyao W Wentao Z Yuhua H. Basic principles of gross ecosystem product (GEP) accounting. J Resour Ecol. (2022) 13:501–10. doi: 10.5814/j.issn.1674-764x.2022.03.014

[10] Ouyang Z Song C Zheng H Polasky S Xiao Y Bateman IJ. Using gross ecosystem product (GEP) to value nature in decision making. Proc Natl Acad Sci. (2020) 117:14593–601. doi: 10.1073/pnas.1911439117, PMID: 32513694 PMC7322027

[11] WHO (2019). Global report on traditional and complementary medicine. WHO. Available online at: https://www.who.int/publications/i/item/978924151536 (Accessed July 1, 2023).

[12] Lindberg K Martvall A Bastos Lima MG Franca CSS. Herbal medicine promotion for a restorative bioeconomy in tropical forests: a reality check on the Brazilian Amazon. Forest Policy Econ. (2023) 155:103058. doi: 10.1016/j.forpol.2023.103058

[13] Scharmer CO Kaufer K. Leading from the emerging future: From ego-system to eco-system economies Berrett-Koehler Publishers (2013).

[14] Dictionary O. E. (2010). Online etymology Dictionary. Available online at: http://www.etymonline.com/index.php?search=suffering&searchmode=none.

[15] Editorial. What is health? Ability Adapt Lancet. (2009) 373:781. doi: 10.1016/S0140-6736(09)60456-6, PMID: 19269498

[16] Mittelmark MB Bauer GF. Salutogenesis as a theory, as an orientation and as the sense of coherence. Handbook Salutogenesis. (2022):11–7. doi: 10.1007/978-3-030-79515-3_3 36122001

[17] Martín-María N Miret M Caballero FF Rico-Uribe LA Steptoe A Chatterji S. The impact of subjective well-being on mortality: a Meta-analysis of longitudinal studies in the general population. Psychosom Med. (2017) 79:565–75. doi: 10.1097/PSY.0000000000000444, PMID: 28033196

[18] Hughes K Bellis MA Hardcastle KA Sethi D Butchart A Mikton C. The effect of multiple adverse childhood experiences on health: a systematic review and meta-analysis. Lancet Public Heal. (2017) 2:e356–66. doi: 10.1016/S2468-2667(17)30118-4, PMID: 29253477

[19] Capon A. Understanding planetary health. Lancet. (2020) 396:1325–6. doi: 10.1016/S0140-6736(20)32150-4

[20] Bircher J Kuruvilla S. Defining health by addressing individual, social, and environmental determinants: new opportunities for health care and public health. J Public Health Policy. (2014) 35:363–86. doi: 10.1057/jphp.2014.19, PMID: 24943659 PMC4119253

[21] Kuruvilla S Sadana R Montesinos EV Beard J Vasdeki JF de Carvalho IA. A life-course approach to health: synergy with sustainable development goals. Bull World Health Organ. (2018) 96:42–50. doi: 10.2471/BLT.17.198358, PMID: 29403099 PMC5791871

[22] Greer SL Falkenbach M Siciliani L McKee M Wismar M Figueras J. From health in all policies to health for all policies. lancet Public Heal. (2022) 7:e718–20. doi: 10.1016/S2468-2667(22)00155-4, PMID: 35907422 PMC9330081

[23] Whitmee S Haines A Beyrer C Boltz F Capon AG de Souza Dias BF. Safeguarding human health in the Anthropocene epoch: report of the Rockefeller Foundation lancet commission onPlanetary health. Lancet. (2015) 386:1973–2028. doi: 10.1016/S0140-6736(15)60901-1, PMID: 26188744

[24] Myers S Frumkin H. Planetary health: Protecting nature to protect ourselves Island Press (2020).

[25] Zinsstag J Kaiser-Grolimund A Heitz-Tokpa K Sreedharan R Lubroth J Caya F. Advancing one human–animal–environment health for global health security: what does the evidence say? Lancet. (2023) 401:591–604. doi: 10.1016/S0140-6736(22)01595-1, PMID: 36682371

[26] WHO (2020). WHO global strategy on health, environment and climate change: The transformation needed to improve lives and wellbeing sustainably through healthy environments. Geneva. Available online at: https://apps.who.int/iris/handle/10665/331959

[27] WHO. Geneva charter. In towards developing WHO’s agenda on well-being. Geneva: World Health Organization (2021).

[28] WHO (2022). Global framework for integrating well-being into public health utilizing a health promotion approach. World Heal. Assem. Available online at: https://apps.who.int/gb/e/e_wha76.html (Accessed June 25, 2023).

[29] Krech R Abdelaziz FB McCartney G Myers SS Boarini R Valentine N. The Geneva charter-realising the potential of a well-being society. Heal Promot J Aust Off J Aust Assoc Heal Promot Prof. (2023) 34:272–5. doi: 10.1002/hpja.735, PMID: 37104508

[30] WHO (2022). WHO draft Framework on achieving Well-being. Geneva. Available online at: https://www.who.int/news-room/articles-detail/call-for-public-comment--who-draft-framework-on-achieving-well-being

[31] Miret M Caballero FF Olaya B Koskinen S Naidoo N Tobiasz-Adamczyk B. Association of experienced and evaluative well-being with health in nine countries with different income levels: a cross-sectional study. Glob Health. (2017) 13:65. doi: 10.1186/s12992-017-0290-0, PMID: 28835255 PMC5568061

[32] López-Otín C Kroemer G. Hallmarks of health. Cell. (2021) 184:33–63. doi: 10.1016/j.cell.2020.11.034 33340459

[33] Indrayan A Vishwakarma G Verma S Sarmukaddam S Tyagi A. Quest for biomarkers of positive health: a review. Indian J Community Med. (2023) 48 10.4103/ijcm.ijcm_480_22 PMC10353687 37469906

[34] Bodeker G Pecorelli S Choy L Guerra R Kariippanon K. Well-being and mental wellness Oxford Research Encyclopedia of Global Public Health (2020).

[35] WHO (2019). Declaration of Astana: Global conference on primary health care: Astana, Kazakhstan, 25 and 26 October 2018. Geneva: World Health Organization. Available online at: https://www.who.int/about/governance/constitution (Accessed June 27, 2023).

[36] Adams J Chung V Dubois J Rodondi P-Y. The significance of traditional and complementary medicine to self-care and addressing Global Health challenges: advancing the commitments of the declaration of Astana through a strong research program. J Integr Complement Med. (2023) 29:337–9. doi: 10.1089/jicm.2023.0004, PMID: 37074342

[37] Priyadarshini P Abhilash PC. Promoting tribal communities and indigenous knowledge as potential solutions for the sustainable development of India. Environ Dev. (2019) 32:100459. doi: 10.1016/j.envdev.2019.100459

[38] Fornasier M. Microcosm and macrocosm in the renaissance. Encyclopedia Renaissance Philosophy. (2022):2185–7. doi: 10.1007/978-3-319-14169-5_949

[39] Tu’itahi S Watson H Egan R Parkes MW Hancock T. Waiora: the importance of indigenous worldviews and spirituality to inspire and inform planetary health promotion in the Anthropocene. Glob Health Promot. (2021) 28:73–82. doi: 10.1177/17579759211062261, PMID: 34931576 PMC8821976

[40] Atuire CA Hassoun N. Rethinking solidarity towards equity in global health: African views. Int J Equity Health. (2023) 22:52. doi: 10.1186/s12939-023-01830-9, PMID: 36964530 PMC10038363

[41] Avicenna. Al Qanun fil Tibb (canon of medicine). New Delhi, India: Jamia Hamdard Printing Press (1988).

[42] Patwardhan B Mutalik G Tillu G. Integrative approaches for health: Biomedical research, Ayurveda and yoga. 1st ed Elsevier Inc (2015).

[43] Dixon M. European congress for integrative medicine. Chair Coll. Med. (2021)

[44] Gaudet T Kligler B. Whole health in the whole system of the veterans administration: how will we know we have reached this future state? J Altern Complement Med. (2019) 25:S7–S11. doi: 10.1089/acm.2018.29061.gau, PMID: 30870023

[45] Montori V. Why we revolt: A patient revolution for careful and kind care. Rosetta Books: (2020).

[46] Patwardhan B Bodeker G. Ayurvedic genomics: establishing a genetic basis for mind-body typologies. J Altern Complement Med. (2008) 14:571–6. doi: 10.1089/acm.2007.0515, PMID: 18564959

[47] Rotti H Raval R Anchan S Bellampalli R Bhale S Bharadwaj R. Determinants of prakriti, the human constitution types of Indian traditional medicine and its correlation with contemporary science. J Ayurveda Integr Med. (2014) 5:167–75. doi: 10.4103/0975-9476.140478, PMID: 25336848 PMC4204287

[48] Rezadoost H Karimi M Jafari M. Proteomics of hot-wet and cold-dry temperaments proposed in Iranian traditional medicine: a network-based study. Sci Rep. (2016) 6:30133. doi: 10.1038/srep30133, PMID: 27452083 PMC4959000

[49] Yoo J Lee E Kim C Lee J Lixing L. Sasang constitutional medicine and traditional Chinese medicine: A comparative overview. Evidence Based Complement. Altern Med. (2012) 2012:1–17. doi: 10.1155/2012/980807 PMC3176432 21941592

[50] Guo R Luo X Liu J Liu L Wang X Lu H. Omics strategies decipher therapeutic discoveries of traditional Chinese medicine against different diseases at multiple layers molecular-level. Pharmacol Res. (2020) 152:104627. doi: 10.1016/j.phrs.2020.104627, PMID: 31904505

[51] Patwardhan B Mashelkar RA. Traditional medicine-inspired approaches to drug discovery: can Ayurveda show the way forward? Drug Discov Today. (2009) 14:804–11. doi: 10.1016/j.drudis.2009.05.009, PMID: 19477288

[52] Patwardhan B. Where lies the future of Ayurveda-inspired drug discovery? Expert Opin Drug Discov. (2023) 18:947–9. doi: 10.1080/17460441.2023.2228201, PMID: 37345376

[53] Shen B. A new golden age of natural products drug discovery. Cell. (2015) 163:1297–300. doi: 10.1016/j.cell.2015.11.031, PMID: 26638061 PMC5070666

[54] Milicic A Reinke S Fergusson J Lindblad EB Thakur A Corby G. Adjuvants, immunomodulators, and adaptogens. Vaccinology Methods Vaccine Res. (2022):223–80. doi: 10.1016/B978-0-323-91146-7.00009-3

[55] Chopra A Tillu G Chuadhary K Reddy G Srivastava A Lakdawala M. Co-administration of AYUSH 64 as an adjunct to standard of care in mild and moderate COVID-19: a randomized, controlled, multicentric clinical trial. PLoS One. (2023) 18:e0282688. doi: 10.1371/journal.pone.0282688, PMID: 36928877 PMC10019690

[56] Prempree P Mungaomklang A Tangkiatkumjai M Phodha T Kwankhao P Chewchuapun K. SARS-CoV-2 clearance from *Andrographis paniculata*, Boesenbergia rotunda, and Favipiravir among mild COVID-19 cases in Klong Prem central prison during Mid-2021: a retrospective study. OSIR J. (2022) 15:131–7. doi: 10.59096/osir.v15i4.262277

[57] Di Francesco A Di Germanio C Bernier M De Cabo R. A time to fast. Science. (2018) 362:770–5. doi: 10.1126/science.aau2095, PMID: 30442801 PMC8504313

[58] Mattson MP Longo VD Harvie M. Impact of intermittent fasting on health and disease processes. Ageing Res Rev. (2017) 39:46–58. doi: 10.1016/j.arr.2016.10.005, PMID: 27810402 PMC5411330

[59] Martino TA Harrington ME. The time for circadian medicine. J Biol Rhythm. (2020) 35:419–20. doi: 10.1177/0748730420946501, PMID: 32746705

[60] Bikomeye JC Balza JS Kwarteng JL Beyer AM Beyer KMM. The impact of greenspace or nature-based interventions on cardiovascular health or cancer-related outcomes: a systematic review of experimental studies. PLoS One. (2022) 17:e0276517. doi: 10.1371/journal.pone.0276517, PMID: 36417344 PMC9683573

[61] Song C Ikei H Miyazaki Y. Physiological effects of nature therapy: a review of the research in Japan. Int J Environ Res Public Health. (2016) 13:781. doi: 10.3390/ijerph13080781, PMID: 27527193 PMC4997467

[62] Hansen MM Jones R. The interrelationship of Shinrin-Yoku and spirituality: a scoping review. J Altern Complement Med. (2020) 26:1093–104. doi: 10.1089/acm.2020.0193, PMID: 32931299

[63] Antonelli M Donelli D Carlone L Maggini V Firenzuoli F Bedeschi E. Effects of forest bathing (shinrin-yoku) on individual well-being: an umbrella review. Int J Environ Health Res. (2022) 32:1842–67. doi: 10.1080/09603123.2021.1919293, PMID: 33910423

[64] Zhang D Lee EKP Mak ECW Ho CY Wong SYS. Mindfulness-based interventions: an overall review. Br Med Bull. (2021) 138:41–57. doi: 10.1093/bmb/ldab005, PMID: 33884400 PMC8083197

[65] Bossert L Arzberger K Dorok F Kern J Stickler C Wunderlich M. The effects of mindfulness-based interventions on telomere length and telomerase activity: a systematic review and Meta-analysis. Mindfulness. (2023) 14:495–509. doi: 10.1007/s12671-023-02075-x, PMID: 39991702

[66] Falkenberg RI Eising C Peters ML. Yoga and immune system functioning: a systematic review of randomized controlled trials. J Behav Med. (2018) 41:467–82. doi: 10.1007/s10865-018-9914-y, PMID: 29429046

[67] Kaelin WG Jr Ratcliffe PJ Semenza GL. For their discoveries of how cells sense and adapt to oxygen availability. Nobel Prize. (2019) 1:9.

[68] Taneja MK. Modified Bhramari pranayama in Covid 19 infection. Indian J Otolaryngol Head Neck Surg. (2020) 72:395–7. doi: 10.1007/s12070-020-01883-0, PMID: 32719737 PMC7239502

[69] Chan AWK Lee A Suen LKP Tam WWS. Tai chi qigong improves lung functions and activity tolerance in COPD clients: a single blind, randomized controlled trial. Complement Ther Med. (2011) 19:3–11. doi: 10.1016/j.ctim.2010.12.007, PMID: 21296261

[70] Wilson D Moloney E Parr JM Aspinall C Slark J. Creating an indigenous Māori-centred model of relational health: a literature review of Māori models of health. J Clin Nurs. (2021) 30:3539–55. doi: 10.1111/jocn.15859, PMID: 34046956 PMC8597078

[71] Reid P Cormack D Paine S-J. Colonial histories, racism and health—the experience of Māori and indigenous peoples. Public Health. (2019) 172:119–24. doi: 10.1016/j.puhe.2019.03.027, PMID: 31171363

[72] Jones R. Climate change and indigenous health promotion. Glob Health Promot. (2019) 26:73–81. doi: 10.1177/1757975919829713, PMID: 30964399

[73] Ijaz N Rioux J Elder C Weeks J. Whole systems research methods in health care: a scoping review. J Altern Complement Med. (2019) 25:S21–51. doi: 10.1089/acm.2018.0499, PMID: 30870019 PMC6447996

[74] Chaturvedi S Patwardhan B. Building bridges for integrative medicine. Lancet Psychiatry. (2016) 3:705–6. doi: 10.1016/S2215-0366(16)30145-6, PMID: 27475762

[75] Tu’itahi S Stoneham M Ratima M Simpson T Signal L Puloka V. Timely and significant call for planetary health promotion. Glob Health Promot. (2019) 26:100–1. doi: 10.1177/1757975919888174 31797730

[76] Chaturvedi S Porter J Gopalakrishna K Abraham L Shankar D Patwardhan B. India and its pluralistic health system – a new philosophy for universal health coverage. Lancet Reg Heal Southeast Asia. (2023) 10:100136. doi: 10.1016/j.lansea.2022.100136, PMID: 36938332 PMC10015266

[77] Ogar E Pecl G Mustonen T. Science must embrace traditional and indigenous knowledge to solve our biodiversity crisis. One Earth. (2020) 3:162–5. doi: 10.1016/j.oneear.2020.07.006

[78] Zhao F Cai L Zang C. Latest developments in international regimes relevant to access and benefit-sharing of genetic resources. Biodivers Sci. (2017) 25:1147–55. doi: 10.17520/biods.2017232

[79] Cahyaningsih R Phillips J Brehm JM Gaisberger H Maxted N. Climate change impact on medicinal plants in Indonesia. Global Ecol Conserv. (2021) 30:e01752. doi: 10.1016/j.gecco.2021.e01752

[80] World Health Organization (1946). Constitution of the World Health Organization. Off. Rec. Wld Hlth Org., 2, 100. Available at: https://apps.who.int/gb/bd/PDF/bd47/EN/constitution-en.pdf?ua=1

